# Supplementary material for: Dynamic transcriptomic profiles of zebrafish gills in response to zinc supplementation
Source: BMC Genomics. 2010 Oct 11;11:553. doi: 10.1186/1471-2164-11-553 (PMC3091702; doi:10.1186/1471-2164-11-553)
Supplement: Additional file 2 — Interactive Direct Interaction Network representing the molecular interactions between zinc, copper, iron, calcium and proteins encoded by transcripts changed by zinc supplementation. Mini web-site containing index.html and hyperlinked pages in subdirectory describing a Direct Interaction Network automatically generated based on curated interactions contained within the proprietary PathwayArchitect database. Ovals represent proteins and the circles symbolize metal ions. Objects are coloured by their abundance in zebrafish at the time-point they were significantly different from the control is a scale from -4 fold (dark green) to +4 fold (dark red). Where significant differences were found at more than one time-point, the colour overlay shows expression at the first instance. Dark blue squares denote 'binding', and light blue squares 'expression'; green squares stand for 'regulation', green diamonds for 'metabolism', and green circles for 'promoter binding'. Arrow heads indicate directionality of the interaction where annotated. All nodes and edges can be further interrogated by selecting the relative area of the image. [file 1471-2164-11-553-S2.zip › PathwayArchitect Zn xs DIN/149495.html]

# PROTEIN: NR2F6

|  |  |
| --- | --- |
| Name | NR2F6 |
| Type | PROTEIN |
| Description | nuclear receptor subfamily 2, group F, member 6 |
| Note | Expressed in anterior pituitary [RGD] |
| Alias | Ovalbumin upstream promoter gamma nuclear receptor |
|  | COUPg |
|  | Erbal2 |
|  | Nr2f6 |
|  | ERBA-related gene-2 |
|  | avian erythroblastic leukemia viral (v-erb-a) oncogene homolog-like 2 |
|  | NR2F6 |
|  | EAR-2 |
|  | Ear2 |
|  | V-erbA-related protein EAR-2 |
|  | AV090102 |
|  | EAR2 |
|  | ovalbumin upstream promoter gamma nuclear receptor rCOUPg |
|  | ERBAL2 |
|  | v-erb-a avian erythroblastic leukemia viral oncogene homolog-like 2 |
|  | COUP-TF3 |


---

|  |  |
| --- | --- |
| GO Component | intracellular |
|  | nucleus |


---

|  |  |
| --- | --- |
| GO ID | GO:0003707 |
|  | GO:0005634 |
|  | GO:0003677 |
|  | GO:0007165 |
|  | GO:0000074 |
|  | GO:0006355 |
|  | GO:0004872 |
|  | GO:0050875 |
|  | GO:0005622 |
|  | GO:0004879 |
|  | GO:0004887 |
|  | GO:0046872 |
|  | GO:0006350 |
|  | GO:0003700 |


---

|  |  |
| --- | --- |
| MIM | MIM:132880 |


---

|  |  |
| --- | --- |
| Connectivity | 59 |


---

|  |  |
| --- | --- |
| Entrez ID | 2063 |
|  | 13864 |
|  | 245980 |


---

|  |  |
| --- | --- |
| Agilent ID | A\_44\_P243231 |
|  | A\_14\_P113819 |
|  | A\_14\_P115030 |
|  | A\_53\_P164873 |
|  | A\_51\_P184353 |
|  | A\_53\_P151223 |
|  | A\_23\_P108326 |
|  | A\_53\_P118266 |
|  | A\_43\_P13369 |


---

|  |  |
| --- | --- |
| Cellular Localization | Nucleus |
|  | Cell |
|  | Organelle |


---

|  |  |
| --- | --- |
| Pathway | Zn xs inventory |
|  | Zn xs DIN |


---

|  |  |
| --- | --- |
| GO Process | signal transduction |
|  | cellular physiological process |
|  | regulation of transcription, DNA-dependent |
|  | transcription |
|  | regulation of progression through cell cycle |


---

|  |  |
| --- | --- |
| UniGene | Rn.25840 |
|  | Mm.28989 |
|  | Hs.466148 |


---

|  |  |
| --- | --- |
| Affymetrix Probeset ID | 104554\_at |
|  | 1218\_at |
|  | 1370380\_s\_at |
|  | 1375112\_at |
|  | 1378564\_at |
|  | 1390066\_at |
|  | 1398826\_s\_at |
|  | 1460647\_a\_at |
|  | 1460648\_at |
|  | 209261\_3p\_s\_at |
|  | 209261\_s\_at |
|  | 209262\_s\_at |
|  | 41310\_f\_at |
|  | 49272\_at |
|  | 57829\_s\_at |
|  | AF003926\_at |
|  | 115270\_at |
|  | g12803666\_3p\_a\_at |
|  | Hs.239752.0.A1\_3p\_a\_at |
|  | Hs.239752.0.A1\_3p\_at |
|  | Hs.239752.0.A1\_3p\_x\_at |
|  | l25674\_s\_at |
|  | rc\_AA801029\_s\_at |
|  | rc\_AI009019\_at |
|  | X12794\_at |
|  | 213354\_s\_at |
|  | 41311\_f\_at |
|  | 41312\_r\_at |
|  | 49275\_g\_at |
|  | Hs.239752.1.A1\_3p\_at |
|  | AA453079\_at |
|  | RC\_T64430\_at |
|  | TC20291\_at |
|  | TC20291\_g\_at |
|  | TC20292\_at |


---

|  |  |
| --- | --- |
| GO Function | ligand-dependent nuclear receptor activity |
|  | thyroid hormone receptor activity |
|  | transcription factor activity |
|  | steroid hormone receptor activity |
|  | receptor activity |
|  | DNA binding |
|  | metal ion binding |


---

|  |  |
| --- | --- |
| Nucleotide | NM\_010150 |
|  | X12794 |
|  | AK171552 |
|  | AK216379 |
|  | AK144475 |
|  | NM\_005234 |
|  | AF003926 |
|  | BC063018 |
|  | AK131941 |
|  | L25674 |
|  | AK215858 |
|  | BC008138 |
|  | AK210900 |
|  | X76654 |
|  | BC084544 |
|  | BC002669 |
|  | AK146779 |
|  | AK190665 |
|  | NM\_139113 |


---

|  |  |
| --- | --- |
| Protein | BAE42523 |
|  | NP\_034280 |
|  | NP\_005225 |
|  | AAA37532 |
|  | CAA31282 |
|  | NP\_620813 |
|  | BAE20887 |
|  | AAB61296 |
|  | CAA54097 |
|  | O09017 |
|  | BAE27426 |
|  | AAH02669 |
|  | AAH08138 |
|  | P43136 |
|  | AAH84544 |
|  | AAH63018 |
|  | P10588 |


---

|  |  |
| --- | --- |
| Organism | Mammal |


---

|  |  |
| --- | --- |
| Location | 8 33.5 cM (Mus musculus) |
|  | chromosome 19, 19p13.1 (Homo sapiens) |
|  | chromosome 16, 16p14 (Rattus norvegicus) |
|  | chromosome 8, 8 33.5 cM, 8 B3.3 (Mus musculus) |


---

|  |  |
| --- | --- |
